# Long-interval intracortical inhibition is similar in people with and without amyotrophic lateral sclerosis

**DOI:** 10.1093/braincomms/fcag091

**Published:** 2026-03-16

**Authors:** Roisin McMackin, Yasmine Tadjine, Narin Suleyman, Eva Woods, Serena Plaitano, Antonio Fasano, Friedemann Awiszus, Orla Hardiman, Richard G Carson

**Affiliations:** Discipline of Physiology, School of Medicine, Trinity Biomedical Sciences Institute, Trinity College Dublin, The University of Dublin, Dublin D02 R590, Ireland; Academic Unit of Neurology, School of Medicine, Trinity Biomedical Sciences Institute, Trinity College Dublin, The University of Dublin, Dublin D02 R590, Ireland; Academic Unit of Neurology, School of Medicine, Trinity Biomedical Sciences Institute, Trinity College Dublin, The University of Dublin, Dublin D02 R590, Ireland; Discipline of Physiology, School of Medicine, Trinity Biomedical Sciences Institute, Trinity College Dublin, The University of Dublin, Dublin D02 R590, Ireland; Department of Neurology, Beaumont Hospital, Dublin D09 V2N0, Ireland; Discipline of Physiology, School of Medicine, Trinity Biomedical Sciences Institute, Trinity College Dublin, The University of Dublin, Dublin D02 R590, Ireland; Academic Unit of Neurology, School of Medicine, Trinity Biomedical Sciences Institute, Trinity College Dublin, The University of Dublin, Dublin D02 R590, Ireland; Academic Unit of Neurology, School of Medicine, Trinity Biomedical Sciences Institute, Trinity College Dublin, The University of Dublin, Dublin D02 R590, Ireland; Department of Orthopaedic Surgery, Otto-von-Guericke University, 39120 Magdeburg, Germany; Academic Unit of Neurology, School of Medicine, Trinity Biomedical Sciences Institute, Trinity College Dublin, The University of Dublin, Dublin D02 R590, Ireland; Department of Neurology, Beaumont Hospital, Dublin D09 V2N0, Ireland; Trinity College Institute of Neuroscience and School of Psychology, Trinity College Dublin, The University of Dublin, Dublin D02 R590, Ireland

**Keywords:** amyotrophic lateral sclerosis, transcranial magnetic stimulation, long-interval intracortical inhibition, threshold tracking, coil orientation

## Abstract

Long-interval intracortical inhibition, measured using transcranial magnetic stimulation, provides a non-invasive measure of spinal inhibition at interstimulus intervals below 100 ms and of GABA-B-mediated motor cortical inhibition at interstimulus intervals of 100–200 ms. To date, only a few small studies have investigated if long-interval intracortical inhibition is affected in amyotrophic lateral sclerosis. None have employed threshold tracking protocols or investigated multiple induced current directions. In this study, we aimed to determine if long-interval intracortical inhibition (i) differs between people with amyotrophic lateral sclerosis and healthy controls; (ii) relates to motor symptom severity, disease duration or survival time in those with amyotrophic lateral sclerosis; or (iii) relates to intracortical facilitation or short-interval intracortical inhibition. Employing automated threshold tracking during paired-pulse transcranial magnetic stimulation of the precentral gyrus, long-interval intracortical inhibition was recorded in 30 people with amyotrophic lateral sclerosis [9 female, 21 male, median (range) age: 63.5 (41–79) years] and 45 healthy controls [16 female, 29 male, median (range) age: 57 (34–76) years]. Long-interval intracortical inhibition was recorded with interstimulus intervals of 50, 100, 150 and 200 ms using posterior-to-anterior induced current (LICI_PA_), and with interstimulus intervals of 150 and 200 ms using anterior-to-posterior induced current (LICI_AP_). In subcohorts of both healthy controls and people with amyotrophic lateral sclerosis, short-interval intracortical inhibition was recorded with interstimulus intervals of 1 and 3 ms using posterior-to-anterior induced current and 3 ms using anterior-to-posterior current. Intracortical facilitation was recorded with an interstimulus interval of 10 ms using posterior-to-anterior induced current. No differences were found between those with and without amyotrophic lateral sclerosis in long-interval intracortical inhibition magnitude (*P* ≥ 0.44, Hedge’s *g* ≤ 0.14) or in the interstimulus interval at which maximal long-interval intracortical inhibition occurs (*P* = 0.68, χ2 = 1.5). In those with amyotrophic lateral sclerosis, no statistically significant correlations were identified between long-interval intracortical inhibition measures and disease duration or functional rating scale scores. Statistically significant positive correlations were observed between LICI_PA_ recorded with 100 and 150 ms interstimulus intervals, and between LICI_PA_ recorded with 150 and 200 ms interstimulus intervals, but not between LICI_PA_ and LICI_AP_ measures or between long-interval intracortical inhibition and short-interval intracortical inhibition or intracortical facilitation. Our findings indicate that disinhibition manifested in this disease is primarily not mediated via changes in the cortical GABA-Bergic or spinal circuitry which underpins long-interval intracortical inhibition measures. As LICI_PA_ and LICI_AP_ measures show minimal covariation, it is possible that these measures are underpinned by distinct aspects of motor cortical inhibition.

## Teaser

Long-interval intracortical inhibition, measured using paired-pulse transcranial magnetic stimulation, provides a non-invasive measure of GABA-Bergic cortical inhibition at interstimulus intervals of 100–200 ms or spinal inhibition at a 50 ms interstimulus interval. McMackin *et al*. report no significant difference in these measures between people with amyotrophic lateral sclerosis and healthy controls.

## Graphical Abstract

**Graphical Abstract:**
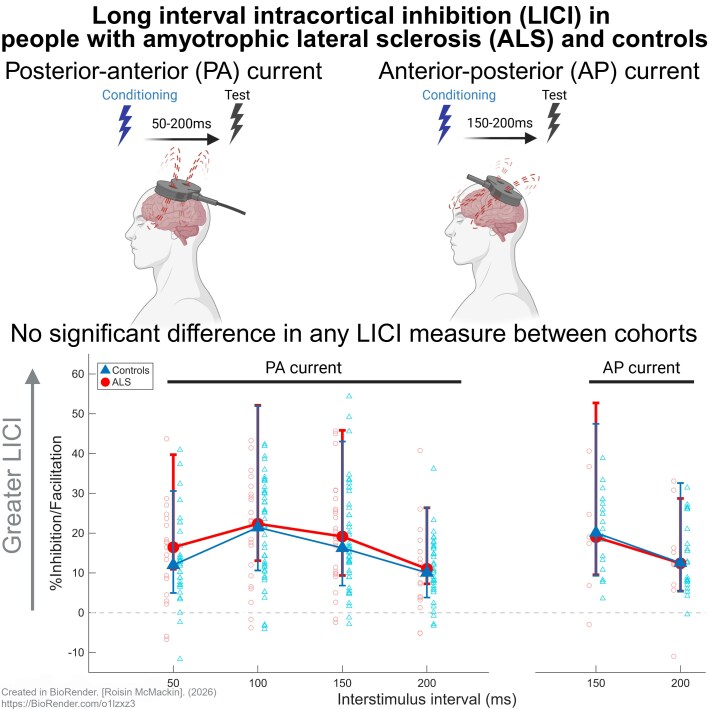

## Introduction

In the search for objective, quantitative and early biomarkers of upper motor neurone dysfunction in amyotrophic lateral sclerosis (ALS), paired-pulse transcranial magnetic stimulation (TMS)-based measures have gathered substantial interest. While inconsistent or small differences have been reported in the case of intracortical facilitation (ICF),^1-3^ short-interval intracortical inhibition (SICI) has consistently been found to be lower (at group level) in cohorts of people with ALS compared to healthy controls or mimic cohorts.^1,3-5^ This difference is found to be greatest where clinical signs of upper motor neurone impairment are absent.^5^ As a result, SICI has been proposed as an early ALS biomarker and has been used to provide supportive evidence for the diagnosis of ALS.^6^ While often discussed under one umbrella term, variants of SICI manifested using different interstimulus intervals (ISIs) are generally considered to reflect distinct underlying processes. Specifically, SICI_1 ms_ (obtained using a 1 ms ISI) has been associated with level of tonic GABAergic activity^7^ and/or refractory activity.^8^ Whereas, it has been proposed that SICI_3 ms_ (obtained using a 3 ms ISI) reflects GABA-Aergic synaptic activity.^9^ The lower SICI evident in ALS (relative to healthy controls) expressed across these ISIs is thus largely considered to reflect disinhibition of upper motor neurones through decline in GABAergic signalling.^10,11^

Despite accelerating use of SICI as an ALS biomarker, long-interval intracortical inhibition (LICI)—another TMS-based measure of intracortical inhibition, is relatively understudied in people with ALS. So called because of the use of a longer ISI compared to SICI, LICI is observed when a motor evoked potential (MEP)-eliciting TMS ‘test’ pulse is preceded by a suprathreshold ‘conditioning’ pulse with an ISI in the range 50–200 ms. As the conditioning stimulus is found to reduce test stimulus-evoked late indirect (I2 and later) waves in epidural spinal recordings of descending volleys,^12,13^ it has been inferred that, for ISIs in the range 100–200 ms, the inhibitory effect occurs at the cortical level. This 100–200 ms ISI range corresponds with the timeframe of GABA-B receptor activity,^14^ and a number of studies have found that LICI recorded with a 100 ms ISI (LICI_100 ms_) is increased following administration of 50 mg of the GABA-B agonist baclofen.^15-17^ This timeframe also corresponds to the window wherein the N100 motor cortical potential evoked by TMS pulses is observed, which peaks at ∼100 ms post-stimulus, which is also enhanced by baclofen (50 mg).^17,18^ On the basis of this collection of evidence, LICI measures across a 100–200 ms ISI range are considered to reflect the degree of GABA-Bergic signalling in the motor cortex. As such, they have been used to infer the effects of disease on GABA-Bergic signalling.^19,20^ Notably, epidural recordings demonstrate that, when an ISI of 50 ms is employed, the indirect waves evoked by the test stimulus are instead boosted by the conditioning stimulus. This is observed despite robust inhibition of test stimulus evoked MEPs.^12^ This suggests that LICI evoked at this shorter interval reflects distinct, spinal-level inhibition.

Of the small number of studies that have investigated LICI in ALS to date, one observed significantly less LICI, when this was averaged across 100 and 150 ms ISIs, in 40 people with ALS compared to 30 healthy controls. It was also reported that the average LICI measure correlated with disease duration.^3^ Another study reported less LICI_100–200 ms_ (but similar LICI_55 ms_) in two small cohorts of people with spinal onset ALS (*n* = 6) and bulbar onset ALS (*n* = 5) compared to healthy controls (*n* = 12).^21^

All studies of LICI in ALS to date have used what is referred to as a posterior-to-anterior (PA) coil orientation. This indicates that the stimulating coil is positioned such that the magnetic pulses induce a flow of electrical current that is directed perpendicularly across the precentral gyrus, proceeding from posterior to anterior.^22^ Studies in healthy controls have indicated that the magnitude of LICI (invoked using ISIs between 160 and 180 ms) is greater when the induced current flows from anterior-to-posterior (AP) across the precentral gyrus, compared to when a PA coil orientation is used.^23^ We recently demonstrated that SICI elicited using a PA (SICI_PA_) and AP (SICI_AP_) coil orientation are not closely correlated. While both measures are lower/absent in people with ALS compared to healthy controls, they are differentially sensitive to the disease state.^1^ With respect to clinical conditions in particular, these studies highlight the value of using more than one coil orientation to derive TMS-based measures. All studies investigating LICI in ALS to date have also used the ‘fixed intensity’ (or sometimes referred to as ‘amplitude-based’) approach to paired-pulse TMS. It has been shown that this approach can be limited by ceiling effects, whereby a cluster of values indicating 100% inhibition are observed. By contrast, a ‘threshold hunting’ or ‘threshold tracking’ approach can be employed to similarly quantify the degree of LICI without suffering these ceiling effects.^24^ This approach has yet to be taken when measuring LICI in ALS.

The aims of this study were to employ a threshold tracking approach to determine whether LICI, recorded across a range of ISIs, differs between people with ALS and healthy controls. In addition, we aimed to determine if the use of an AP-induced current direction affects the magnitude of any observed differences in LICI between these cohorts. Finally, we aimed to characterize the relationship between LICI measures recorded at different ISIs, or with different induced current directions, and other TMS-derived measures which have been more extensively studied in ALS, including SICI, ICF and resting motor threshold (RMT).

## Materials and methods

### Recruitment and eligibility criteria

Ethical approval was obtained from the ethics committee of St. James’s Hospital (REC reference: 2017-02). All participants were over 18 years of age and could provide informed written or verbal consent (in the presence of two witnesses) prior to participation. Data were collected between 2019 and 2024 in the Wellcome-HRB Clinical Research Facility of St. James’ Hospital, Dublin. Except for preregistration, all work was performed in accordance with the Declaration of Helsinki. All participants were screened according to the TMS screening questionnaire of Rossi *et al*.^25^ and excluded if contraindications to TMS were identified. Those in the ALS cohort were diagnosed with Possible, Probable or Definite ALS in accordance with the El Escorial Revised Diagnostic Criteria. Controls included neurologically healthy individuals recruited from an existing population-based cohort of individuals who registered interest in volunteering for neurological research. Of those who underwent TMS, 15 people with ALS and 2 healthy controls were excluded from this analysis due to RMT exceeding 83% of maximum stimulator output (%MSO) in both hemispheres. As such application of conditioning stimuli at 120% of RMT (e.g. >100%MSO), required to record LICI, was not possible in these individuals. A total of 30 people with ALS and 45 healthy controls participated (Table 1, *n* per measurement in Table 2), recruited between 2017 and 2024. Sample sizes exceeded those (*n*_control_ = 16, *n*_ALS_ = 10) required to replicate the prior reported difference in LICI_PA-100–150 ms_ in ALS compared to healthy controls (Cohen’s *d* ≈ 1.2^3^) with an allocation ratio (*n*_control_/*n*_ALS_) of 1.5. Handedness was determined on the basis of Edinburgh Handedness Inventory, where a Laterality Quotient < 0 indicates left handedness.^26^ Two people with ALS were taking amitryptiline, four people with ALS were taking baclofen, and all except one of the individuals with ALS was taking riluzole (all for more than 12 weeks, except for one individual who commenced riluzole treatment 7 weeks prior).

**Table 1:** Summary of statistics for ALS and healthy control cohorts

Cohort	Controls (*n* = 45)	ALS (*n* = 30)	*P*
Sex (f/m)	16/29	9/21	0.62
Median age in months [range]	57 [34–76]	63.5 [41–79]	0.02
Handedness (right/left)	44/1	28/2	0.34
Hemisphere to which TMS was applied (right/left)	1/44	5/25	0.02
Time since participant-estimated first symptom onset in months median [range]	N/A	22 [7–96]	N/A
Time since diagnosis median [range] in months	N/A	8 [2–80]	N/A
ALSFRS-R total score median [range] (available for ALS cohort *n* = 24)	N/A	41 [17–47]	N/A
∂ALSFRS-R (available for ALS cohort *n* = 24)	N/A	0.32 [0.02–4.43]	N/A
Region of onset (spinal/bulbar/respiratory)	N/A	26/2/2	N/A
Site/side of first symptom onset for spinal onset (upper left/lower left/upper right/lower right/upper bilateral/lower bilateral)	N/A	2/5/5/6/4/4	N/A
El Escorial criterion (possible/probable/definite/lab-supported probable)	N/A	4/8/6/1	N/A

*P* values for sex and handedness were determined by χ^2^ testing. Age *P* value was determined by sign rank testing. ALSFRS-R—Revised ALS functional rating scale. ∂ALSFRS-R is calculated as: (48-ALSFRS-R)/months since symptom onset. Record of El Escorial category at time of diagnosis was retrievable for 19 out of 30 people with ALS.

**Table 2:** Summary statistics for each TMS parameter recorded

	Controls				ALS					
Measure	*N*	CTT > 100	CS > 100	Median	*N*	CTT > 100	CS > 100	Median	Group *P* (*g* [95% CI])	AUROC (95% CI)
RMT_PA_	45	N/A	N/A	52.24	30	N/A	N/A	55.84	0.23 (0.29 [−0.22–0.77])	0.58 [0.44–0.72]
RMT_AP_	43	1	N/A	68.38	28	1	N/A	70.29	0.68 (0.15 [−0.35–0.61])	0.53 [0.39–0.66]
THT_PA_	45	N/A	N/A	57.40	30	N/A	N/A	62.87	0.25 (0.22 [−0.18–0.68])	0.58 [0.45–0.71]
THT_AP_	37	4	N/A	72.87	23	5	N/A	74.61	0.22 (0.34 [−0.15–0.85)	0.59 [0.45–0.74]
LICI_PA-50 ms_	32	2	N/A	11.99	19	0	N/A	16.45	0.44 (0.14 [−0.40–0.70])	0.57 [0.39–0.74]
LICI_PA-100 ms_	43	1	N/A	21.45	22	5	N/A	22.36	1 (0.00 [−0.53–0.51])	0.50 [0.35–0.64]
LICI_PA-150 ms_	44	1	N/A	16.28	25	3	N/A	19.17	0.68 (0.08 [−0.40–0.60])	0.53 [0.39–0.67]
LICI_PA-200 ms_	35	0	N/A	10.06	18	1	N/A	11.03	0.90 (0.04 [−0.61–0.64])	0.51 [0.33–0.69]
LICI_PA-Mean_	32	N/A	N/A	8.37	14	N/A	N/A	11.16	0.62 (0.03 [−0.59–0.64])	0.55 [0.35–0.71]
LICI_PA-Max_	32	N/A	N/A	14.04	14	N/A	N/A	21.15	0.84 (0.05 [−0.54–0.64])	0.52 [0.33–0.70]
LICI_AP-150 ms_	22	3	5	20.09	7	4	3	19.01	0.94 (0.31 [−0.11–1.04])	0.51 [0.22–0.82]
LICI_AP-200 ms_	23	2	5	12.53	10	1	3	12.42	0.92 (−0.03 [−0.82–0.94])	0.51 [0.26–0.72]
LICI_AP-Mean_	21	N/A	N/A	10.71	7	N/A	N/A	9.45	0.87 (−0.01 [−1.22–1.14])	0.52 [0.23–0.80]
LICI_AP-Max_	21	N/A	N/A	14.42	7	N/A	N/A	13.82	1 (−0.02 [−1.18–0.98])	0.50 [0.22–0.77]

Median values for RMT and THT are expressed in % of maximum stimulator output (%MSO). Median values for LICI are expressed in %inhibition, calculated according to Equation (1). Group *P* (d) column values refer to *P* value and Hedge’s *g* for the comparison of ALS and control values. 95% CI—95% confidence interval. CTT > 100—numbers of datasets excluded due to the conditioned threshold target (CTT) exceeding 100% MSO. CS > 100—numbers of datasets excluded due to the required conditioning stimulus exceeding >100% MSO. Measure subscript text indicate direction of induced current and interstimulus interval employed. PA—posteroanterior. AP—anteroposterior. *P* values listed are uncorrected. Mean/Max—mean/maximum inhibition values across 100, 150 and 200 ms ISIs. Those coefficient values with corrected *P* values (at 5% false discovery rate) < 0.05 are emboldened. AUROC—area under the receivership operating characteristic curve.

LICI = long-interval intracortical inhibition; RMT = resting motor threshold; THT = threshold hunting target; *N* = numbers of datasets recorded.

### Transcranial magnetic stimulation

We first applied single- and paired-pulse TMS over the motor cortex contralateral to the dominant hand, with the coil oriented so as to induce PA directed current across the precentral gyrus. Concurrently, EMG was recorded in abductor pollicis brevis of the dominant hand (APB). In three right-handed people with ALS, TMS was applied to the right hemisphere and EMG was recorded in the left APB. For two of these individuals, MEPs of sufficient amplitude could not be obtained from the right APB, attributed to muscle atrophy. For the third individual, the non-dominant hand was selected to avoid potential influence of residual inflammation from a recent distal fracture in the right arm. This participant did not report any discomfort during participation. In total, LICI was recorded in APB on the side of symptom onset for eight people with spinal-onset ALS, with three of these individuals having onset in the upper limb. Hardware, software, hotspotting and coil positioning protocols and EMG recording parameters employed were exactly as described in McMackin *et al*.^2^

### Threshold tracking method

A threshold tracking-based approach was employed to measure LICI, using a fully automated parameter estimation by sequential testing (PEST)-based protocol. A detailed description of this protocol is provided in McMackin *et al*.^1^ This PEST protocol was implemented using a combination of MATLAB (R2016a, MathWorks Ltd, UK) and Signal (Version 7.0, Cambridge Electronic Design Ltd, UK) scripts, which deliver a maximum likelihood PEST protocol, as is widely utilized in manual form via the TMS Motor Threshold Assessment Tool (MTAT 2.0, created by Prof. Friedemann Awiszus, available at: https://www.clinicalresearcher.org/software.htm). These automated scripts measure the evoked MEP amplitude (peak-to-peak amplitude 15–50 ms following test pulse delivery), input this value into the maximum likelihood PEST algorithm, identify the amended threshold intensity recommendation, and adjust stimulator output to deliver the next pulse at this intensity. This automated approach prevents the experimenter from needing to manually read and input EMG measurements to the algorithm between pulses, limiting human error and allowing us to apply pulses with an inter-trial interval of 5 s (+/−10% time jitter). Employing this PEST-based approach, for each participant we determined the stimulation intensity (in %MSO) which would, in 50% of cases, evoke an MEP with peak-to-peak amplitude of at least 50 µV (RMT_PA_) and 200 µV (threshold hunting target, THT_PA_). We subsequently recorded LICI by repeating the procedure for recording THT_PA_, but with each test stimulus preceded by a conditioning stimulus fixed at 120% of RMT_PA_. In this case, the test stimulation intensity which, in 50% of cases, evokes an MEP with peak-to-peak amplitude of at least 200 µV is referred to as the conditioned threshold target (CTT) and LICI is measured as:


(1) $$\mathrm{LICI}(\text{\%})=\;\left(\frac{\mathrm{CTT}\;-\;\mathrm{THT}}{\mathrm{THT}}\;\times 100\text{\%}\;\right)\;$$

Larger positive LICI values indicate greater inhibition. Four LICI measures were recorded, using ISIs of 50 (LICI_PA-50 ms_), 100 ms (LICI_PA-100 ms_), 150 ms (LICI_PA-150 ms_) and 200 ms (LICI_PA-200 ms_). Previously, for ISIs of 160–180 ms but not 100 ms,^23^ LICI has been found to be significantly greater when recorded with AP-induced currents compared to when PA-induced currents are employed. On this basis, as additional measures (e.g. SICI, ICF) were also recorded during this research visit, we opted to omit LICI _AP-50–100 ms_ measurements to limit study session durations. At the same hotspot location, with the coil oriented so as to induce AP directed current across the precentral gyrus, the same protocol was employed to measure RMT_AP_, THT_AP_, LICI_AP-150 ms_ and LICI _AP-200 ms_. The order in which LICI measurements were recorded, across both coil orientations, was randomized. Each measurement was independently recorded (i.e. the PEST algorithm was restarted for each measure without carry over of information from the prior measure), to prevent any potential bias of the tracking algorithm by prior measurements. To monitor relaxation throughout threshold tracking, root mean square amplitude in the 200 ms immediately preceding stimulation was measured, and where this amplitude exceeded 10 µV, the MEP was not passed to the PEST algorithm and the pulse trial was repeated. In the case of a minority (*n*_control_ = 9, *n*_ALS_ = 8) of later participants, due to time availability, a reduced protocol was employed where LICI_AP_ measures and LICI_PA-50 ms_ and LICI_PA-200 ms_ were not recorded. Where CTT exceeded 100%MSO, LICI could not be definitively measured. Numbers of datapoints for each measure, and numbers in whom CTT > 100%MSO, are outlined in Table 2. Measurements of ICF_PA_ (10 ms ISI, 70% RMT_PA_ conditioning stimulus), SICI_PA_ (1 and 3 ms ISI, 70% RMT_PA_ conditioning stimulus) and SICI_AP_ (3 ms ISI, 70% RMT_AP_ conditioning stimulus), recorded as outlined in McMackin *et al*., 2024, were also available for a subset of all participants. Most of these measurements, recorded on the same day as those reported here, are those included in our prior study. Specifically there is overlap in the cohort reported in McMackin *et al*., 2024 and this study for 37 healthy controls and 24 people with ALS for ICF_PA_, for 34 healthy controls and 23 people with ALS for SICI_PA-1 ms_, for 34 healthy controls and 23 people with ALS for SICI_PA-3 ms_, and for 18 healthy controls and 9 people with ALS for SICI_AP-3 ms_. Overall, for all participants where SICI, ICF and LICI measures were recorded within one session, TMS recordings from beginning hotspotting to final measurement took approximately 1.5 h, including regular breaks between measurements.

### Maximum compound muscle action potentials

Maximum compound muscle action potentials (mCMAPs) were recorded in the muscle from which MEPs were recorded during TMS, for 21 people with ALS and 34 healthy controls who provided RMT_PA_ data. Of this cohort, 29 healthy controls and 19 people with ALS were included in the CMAP analysis reported in McMackin *et al*., 2024. All EMG recording apparatus was identical to that used for MEP recording. Electrical stimulation was delivered over the median nerve by a Digitimer DS7A stimulator (Digitimer Ltd, Welwyn Garden City, UK) at either the elbow or the wrist. Latency and maximum peak-to-peak amplitude were examined within the 2–30 ms window following test stimulation. A detailed outline of CMAP recording and signal procedures is outlined in McMackin *et al*., 2024.

### Statistical analysis

All statistical analyses were applied in MATLAB R2024a. To account for multiple comparisons, *P* values were corrected within analyses across LICI measures to obtain a positive false discovery rate (FDR) below 5% by the Benjamini–Hochberg method.^27^ Corrected *P* values below 0.05 were considered significant. Shapiro–Wilk^28^ tests indicated that some LICI measures did not conform to a normal distribution, therefore, non-parametric statistics were employed.

#### Cohort differences

Ratio of male to female sex, ratio of left to right handedness, and ratio of left to right hemisphere stimulated, were compared between cohorts by χ2 testing. Ages of healthy controls and those with ALS were compared by Mann–Whitney U-test. To test if the expected inhibitory effects were reliably evoked, sign rank testing of control values (compared to zero) was performed for each LICI measure. Mann–Whitney U testing was employed to test the significance of differences in TMS measures between cohorts as some measures were deemed to have distributions that deviated significantly from normality by Shapiro–Wilk testing (*P* < 0.05). As epidural recording studies indicate that the physiological underpinnings of LICI_50 ms_ and LICI_100–200 ms_ differ,^12,13^ mean and maximum LICI_PA_ values were calculated excluding LICI_PA-50 ms_. Maximum and mean LICI_PA_ was determined only in those from whom LICI_PA_ measures were recorded at 100, 150 and 200 ms ISIs. Maximum LICI_AP_ was determined in those from whom both LICI_AP_ measures were recorded. To compare between cohorts the ratio of individuals who displayed maximum LICI_PA_ at each ISI, a χ2 analysis (4 ISIs × 2 cohorts) was performed. These ratios include only those where LICI_PA_ values were recorded at all four ISIs and all CTT values were <100%MSO (ALS *n* = 14, control *n* = 32) or only one CTT value was >100%MSO, and therefore represents the ISI which evokes the most potent LICI (ALS *n* = 2 at ISI = 100 ms). To quantify the potential of paired-pulse TMS measures to discriminate people with ALS from healthy controls, area under the receiver operating characteristic curve (AUROC) was calculated for each TMS parameter for each ISI and coil orientation used.

To verify our prior report of CMAP characteristics in an overlapping cohort,^1^ we repeated the linear mixed effects modelling of group and site of stimulation effects, including their interaction, as well as age, as follows (according to Wilkinson notation):


$$\begin{array}{c} \mathrm{Median}\;\;\mathrm{Latency}\;=\;\mathrm{Group}\;*\;\mathrm{Site}\;+\;\mathrm{Age}\;+\;(\mathrm{Group}\;*\;\mathrm{Site}\;|\;\mathrm{Subject})\\ \mathrm{Maximum}\;\;\mathrm{Amplitude}\;=\;\mathrm{Group}\;*\;\mathrm{Site}\;+\;\mathrm{Age}\\ \;\;\;+\;(\mathrm{Group}\;*\;\mathrm{Site}\;|\;\mathrm{Subject}) \end{array}$$

On the basis that Cohen’s *d* is calculated as group mean difference divided by pooled standard deviation when simply comparing groups,^29^ Cohen’s *d* values for Site (i.e. location of stimulation) and Group were estimated as the coefficient values (corresponding to the mean difference) divided by the standard deviation of the residuals. The 95% confidence interval for this value was calculated by repeating this calculation for the upper and lower 95% confidence interval limits of the coefficient values, calculated by the fitlme function of MATLAB R2016a.

Effect size was measured by Hedge’s *g*.^30^ Values and confidence intervals for Hedge’s *g* and AUROC values were calculated via the ‘Measures of Effect Size’ MATLAB toolbox of Hentschke and Stüttgen,^31^ applying bootstrapping 1000 times.

#### Correlations to age and clinical metrics

Spearman’s correlation analysis was performed to determine the association between each LICI measure and (i) age and, in those with ALS, (ii) disease duration (time since self-reported first symptom onset), (iii) revised ALS functional rating scale (ALSFRS-R) total score, (iv) ALSFRS-R upper limb associated sub scores (handwriting, utensil handling, dressing and hygiene) and (v) mCMAP in right APB (measurement protocol detailed in McMackin *et al*., 2024). ALSFRS-R score was recorded within two months of study participation for 20 people with ALS and scores for an additional 4 people with ALS were estimated based on interpolation of two ALSFRS-R measures taken before and after participation. Correlations between ALSFRS-R and LICI_AP_ measures are not reported due to limited paired data (*n* = 5).

#### Correlations between TMS measures

We investigated correlations between pairs of LICI measures of matching coil orientation or ISI, as well as between LICI measures and (where coil orientation was matching) ICF_PA_, SICI_PA_ and SICI_AP_. As THT is a common denominator in the calculation of all paired-pulse measures according to Equation (1), this may contribute (through mathematical coupling) towards observed positive correlations between these measures. Normalization of the conditioning effect relative to THT [i.e. as in Equation (1)] is typical in TT-TMS studies,^5,23,32^ carrying over the normalization convention employed in fixed intensity TMS studies.^33^ In the case of normalizing TT-TMS measures, the assumption implicit in previous methods is that a specific increase in threshold with addition of a conditioning stimulus reflects a lesser degree of inhibition in those individuals with higher thresholds compared to those with lower thresholds. However, several studies have failed to demonstrate a significant association between recruitment curve slope and either RMT or the S_50_ curve parameter (the stimulation intensity which evokes an average response of half maximum amplitude, which shows strong correlation to RMT).^34-36^ This suggests that the assumption of a (linear) relationship between resting threshold and the increase in stimulation intensity required to overcome an inhibitory conditioning effect may be unfounded. In summary, when calculating measures of association (such as correlation coefficients) the standard method of normalization—which can inflate estimates due to mathematical coupling, may well be unnecessary. Therefore, in order to avoid spurious inflation of correlation coefficients, when examining the relationship between paired pulse TMS measures, we re-calculated inhibition/facilitation for each measure without the usual denominator as follows:


(2) LICI(%MSO)=(CTT−THT)

These ‘non-normalized’ measures were employed in the case of all correlations between paired pulse TMS measures. To account for a potential mediating influence of variations in THT, Spearman’s partial correlations were employed, i.e. to regress out any effect of THT. Spearman’s correlation analyses were also performed to investigate the relationship between THT and each LICI measure [calculated as per Equation (2)]. Correlations were performed on the overall cohort, as well as for ALS and control cohorts separately, with the exception of correlations between LICI_AP_ and SICI_AP_ in ALS, due to small numbers of paired measures (*n* = 4).

## Results

### Comparison of people with ALS to healthy controls

**Figure 1:**
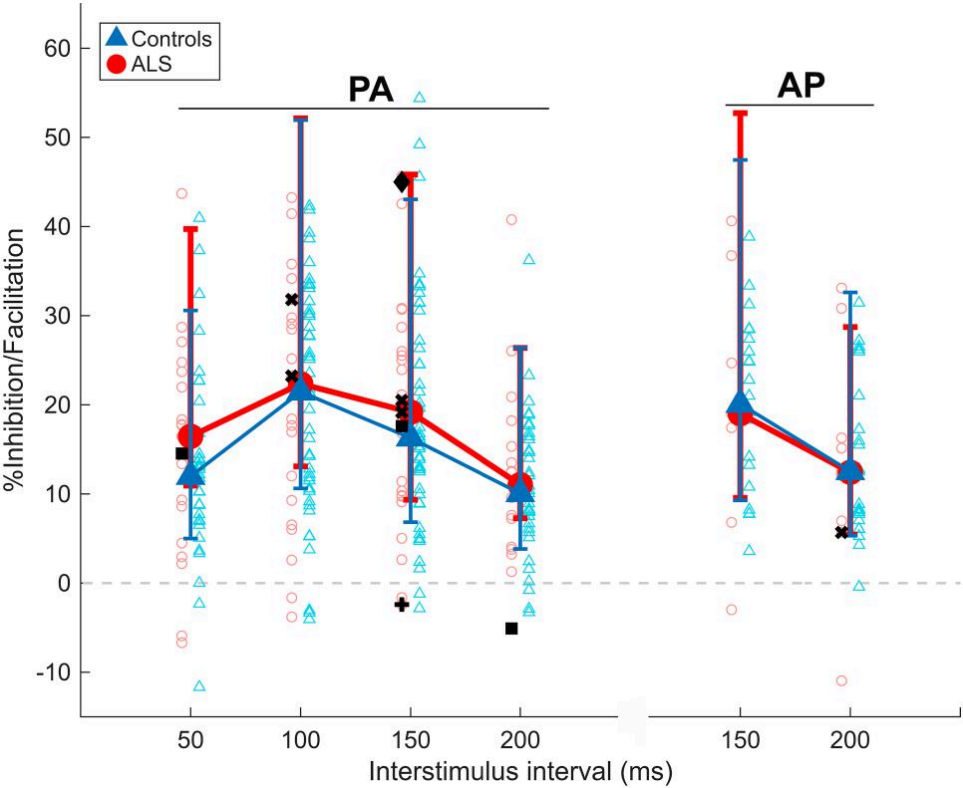
**Long-interval intracortical inhibition median values for those with ALS (red circles) and healthy controls (blue triangles) across orientations and interstimulus intervals tested.** Error bars illustrate interquartile ranges. Sample sizes for each measure are reported in Table 2 (*N* = 22–44 for controls, *N* = 7–25 for people with ALS). Positive *y*-axis values reflect an inhibitory effect of conditioning on the corticospinal tract. Negative *y-*axis values reflect a facilitatory effect of conditioning on the corticospinal tract. Open circles—individual values from ALS cohort. Open triangles—individual values from control cohort. **+**—LICI value of individual with ALS taking amitryptiline. **x**—LICI value of individuals with ALS taking baclofen. ■—LICI value of individual with ALS taking baclofen and not taking riluzole. ◆—LICI value of individual with ALS taking baclofen and amitriptyline. PA—posterior-to-anterior induced current. AP—anterior-to-posterior induced current.

To confirm the validity of the protocol employed, we established that significant LICI was evoked in the control cohort for all coil orientations and ISIs (*P* ≤ 3.09 * 10^−5^). No statistically significant difference in RMT, THT or LICI was observed between people with ALS and healthy controls, for any ISI or coil orientation tested, or for mean or maximum values across ISIs (for all LICI measures: *P* = 0.44–1, Hedge’s *g* = −0.03–0.31, outlined in full in Table 2). Median values and interquartile ranges for each LICI measure for each cohort are illustrated in Fig. 1. These findings did not change for any measure upon exclusion of those taking amitriptyline (for LICI_PA-150 ms_: *P* = 0.64, Hedge’s *g* = 0.07) or baclofen (for all LICI_PA_ measures: *P* = 0.84–0.99, Hedge’s *g* = −0.06–0.14) or those where the non-dominant hemisphere was tested (for all LICI measures: *P* = 0.13–0.98, Hedge’s *g* = 0–0.34). Individual values of these measurements from those taking baclofen or amitriptyline are highlighted as symbols in Fig. 1. Similarly, no statistically significant differences between cohorts were identified when LICI was calculated according to Equation (2). For LICI_PA_ measures, we also found no significant differences between healthy controls and those individuals with spinal-onset ALS where LICI was recorded in the hand on the side of symptom onset (*P* = 0.06–0.52), with the only trend (*P* = 0.06) observed being modestly higher LICI_PA-100 ms_ in this ALS subcohort compared to healthy controls. This analysis was not performed for LICI_AP_ measures due to limited numbers of LICI_AP_ measures in this subcohort. All LICI values measured on or opposite the side of symptom onset are individually shown in Supplementary Fig. 1.

The ISI at which the potency of LICI was maximal did not differ reliably between healthy controls (50 ms = 14, 100 ms = 4, 150 ms = 11, 200 ms = 3) and people with ALS (50 ms = 6, 100 ms = 4, 150 ms = 4, 200 ms = 2) (χ2 = 1.5, *P* = 0.68). Including the four individuals (ALS *n* = 2, control *n* = 2) whose LICI_PA_ CTT exceeded 100%MSO at two ISIs—and repeating the analysis with maximum LICI_PA_ considered to occur at either ISI for each—did not alter the results (χ2 = 0.65–3.90, *P* = 0.27–0.89). A significant negative correlation between the ALSFRS-R cutting food and handling utensils subscore and THT_PA_ was identified (rho = −0.69, *P*_uncorrected_ = 1.7 * 10^−4^). No significant correlations were observed between LICI [with any coil orientation or ISI, calculated according to Equations (1) or (2)] and age, ALSFRS-R subscores or total score or disease duration at a 5% FDR.

In keeping with our prior analysis of an overlapping cohort,^1^ CMAP latency was confirmed to be significantly greater in those with ALS (group effect *P* = 0.038, *d* [95% confidence interval] = 0.29 [0.017–0.56]) with significantly smaller mCMAP amplitude (group effect *P* = 0.007, *d* [95% confidence interval] = −0.380 [−0.651–−0.109]).

### Correlations between LICI measures

**Figure 2:**
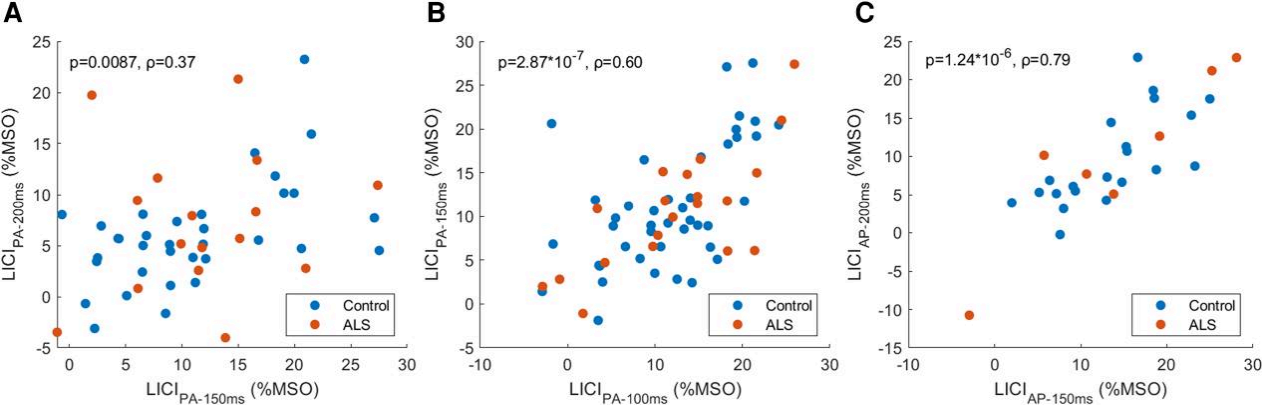
**Scatter plots illustrating correlations between TMS-based measures that were deemed significant following multiple comparison correction.** Data points represent LICI values for each healthy control (blue) and individual with ALS (orange), measured using the coil orientation and interstimulus interval noted in axis labels. ρ and *P* values determined by Spearman’s correlation analyses. Stated *P* values are uncorrected. For subfigure **A**, control *N* = 42, ALS *N* = 20, for subfigure **B**, control *N* = 21, ALS *N* = 7, for subfigure **C**, control *N* = 34, ALS *N* = 16. AP—anterior-to-posterior induced current. PA—posterior-to-anterior induced current. LICI—long-interval intracortical inhibition. %MSO—percentage of maximum stimulator output.

Statistics for correlations between LICI measures [calculated according to Equation (2)] are outlined in Table 3. Statistically significant positive correlations were observed between LICI_PA-100 ms_ and LICI _PA-150 ms_, between LICI_PA-150 ms_ and LICI_PA-200 ms_ and between LICI_AP-150 ms_ and LICI_AP-200s_ (Fig. 2A–C). Similar relationships were observed where each cohort was considered separately, with the exception of the correlation between LICI_PA-150 ms_ and LICI_PA-200 ms_, whereby those with ALS showed a positive but non-reliable correlation (ρ = 0.37, *P* = 0.64), although this sample was limited (*n* = 7). No statistically significant correlations were observed between LICI_PA_ recorded at 50 ms ISI to LICI recorded at 100 ms, 150 ms or 200 ms, or between LICI_PA_ and LICI_AP_ values recorded at matching ISIs. Similar findings were observed if LICI was calculated according to Equation (1).

**Table 3:** Correlation statistics between LICI measures for combined ALS and control cohorts

LICI measure 1		LICI measure 2						
Current direction	ISI	Current direction	ISI	ρ	*P*	*N*_total_	*N*_c_	*N*_ALS_
PA	50	PA	100	0.27	0.07	46	32	14
PA	50	PA	150	0.20	0.17	48	32	16
PA	50	PA	200	0.03	0.83	50	32	18
**PA**	**100**	**PA**	**150**	**0**.**60**	2.87*10^−7^	**62**	**42**	**20**
PA	100	PA	200	0.21	0.16	47	33	14
**PA**	**150**	**PA**	**200**	**0**.**37**	**0**.**0087**	**50**	**34**	**16**
AP	150	PA	150	0.13	0.51	29	22	7
AP	200	PA	200	0.20	0.28	33	23	10
**AP**	**150**	**AP**	**200**	**0**.**79**	1.24*10^−6^	**28**	**21**	**7**

LICI measures are calculated as the difference between conditioned and unconditioned thresholds [Equation (2)]. *P* and ρ values were determined by Spearman’s correlation analyses. Stated *P* values are uncorrected. Emboldened text highlight where correlations were deemed significant when corrected to a 5% FDR. Empty boxes are those where a correlation was not performed as neither coil orientation nor ISI was common across measures, or in the case of autocorrelation.

AP = anterior-to-posterior induced current. PA = posterior-to-anterior induced current. ISI = interstimulus interval *N*_total_ = number of datapoints across cohorts. *N*_C_ = number of control datapoints. *N*_ALS_ = number of ALS datapoints.

### Correlation between LICI and other TMS measures

Statistics for correlations between LICI measures [calculated according to Equation (2)] and other TMS measures are outlined in Table 4. No significant correlations were observed between LICI and THT, SICI or ICF across coil orientations. While modest positive correlations were observed between LICI_PA-150 ms_ and SICI_PA_ measures (SICI_PA-1 ms_: ρ = 0.31, *P* = 0.02, SICI_PA-1 ms_: ρ = 0.27, *P* = 0.04), these correlations were not deemed statistically significant at a 5% FDR. No reliable differences were observed at a 5% FDR when people with ALS and healthy controls were analysed separately.

**Table 4:** Correlation statistics between LICI measures and other TMS-based measures for combined ALS and control cohorts

LICI:	PA-50 ms	PA-100 ms	PA-150 ms	PA-200 ms	PA-max	PA-mean	AP-150 ms	AP-200 ms	AP-max	AP-mean
**THT_PA_**	ρ = −0.24	ρ = −0.02	ρ = 0.02	ρ = −0.04	ρ = 0.00	ρ = 0.00				
	*P* = 0.09	*P* = 0.88	*P* = 0.89	*P* = 0.76	*P* = 0.98	*P* = 0.96				
	*N*_c_ = 32	*N*_c_ = 43	*N*_c_ = 44	*N*_c_ = 35	*N*_c_ = 32	*N*_c_ = 32				
	*N*_ALS_ = 19	*N*_ALS_ = 22	*N*_ALS_ = 25	*N*_ALS_ = 18	*N*_ALS_ = 14	*N*_ALS_ = 14				
**SICI_PA-1 ms_**	ρ = 0.22	ρ = 0.20	ρ = 0.31	ρ = 0.28	ρ = 0.27	ρ = 0.30				
	*P* = 0.16	*P* = 0.13	*P* = 0.02	*P* = 0.07	*P* = 0.09	*P* = 0.06				
	*N*_c_ = 28	*N*_c_ = 38	*N*_c_ = 39	*N*_c_ = 29	*N*_c_ = 28	*N*_c_ = 28				
	*N*_ALS_ = 16	*N*_ALS_ = 19	*N*_ALS_ = 20	*N*_ALS_ = 15	*N*_ALS_ = 12	*N*_ALS_ = 12				
**SICI_PA-3 ms_**	ρ = −0.12	ρ = 0.23	ρ = 0.27	ρ = 0.17	ρ = 0.04	ρ = 0.20				
	*P* = 0.46	*P* = 0.10	*P* = 0.04	*P* = 0.29	*P* = 0.80	*P* = 0.24				
	*N*_c_ = 28	*N*_c_ = 37	*N*_c_ = 38	*N*_c_ = 29	*N*_c_ = 28	*N*_c_ = 28				
	*N*_ALS_ = 15	*N*_ALS_ = 18	*N*_ALS_ = 19	*N*_ALS_ = 14	*N*_ALS_ = 11	*N*_ALS_ = 11				
**ICF_PA-10 ms_**	ρ = 0.11	ρ = 0.14	ρ = 0.22	ρ = 0.20	ρ = 0.17	ρ = 0.28				
	*P* = 0.48	*P* = 0.29	*P* = 0.10	*P* = 0.19	*P* = 0.30	*P* = 0.08				
	*N*_c_ = 30	*N*_c_ = 40	*N*_c_ = 42	*N*_c_ = 32	*N*_c_ = 30	*N*_c_ = 30				
	*N*_ALS_ = 15	*N*_ALS_ = 18	*N*_ALS_ = 19	*N*_ALS_ = 14	*N*_ALS_ = 11	*N*_ALS_ = 11				
**THT_AP_**							ρ = −0.07	ρ = −0.10	ρ = 0.07	ρ = 0.05
							*P* = 0.74	*P* = 0.58	*P* = 0.74	*P* = 0.81
							*N*_c_ = 22	*N*_c_ = 23	*N*_c_ = 21	*N*_c_ = 21
							*N*_ALS_ = 7	*N*_ALS_ = 10	*N*_ALS_ = 7	*N*_ALS_ = 7
**SICI_AP-3 ms_**							ρ=0.18	ρ=0.11	ρ=0.18	ρ=0.19
							*P* = 0.46	*P* = 0.63	*P* = 0.47	*P* = 0.44
							*N*_c_ = 16	*N*_c_ = 17	*N*_c_ = 16	*N*_c_ = 16
							*N*_ALS_ = 4	*N*_ALS_ = 4	*N*_ALS_ = 4	*N*_ALS_ = 4

Column headings indicate [induced current direction]-[interstimulus interval] used to record LICI. All paired-pulse measures are calculated as the difference between conditioned and unconditioned thresholds [Equation (2)]. *P* and ρ values were determined by Spearman’s correlation analyses. Stated *P* values are uncorrected. Empty boxes are those where a correlation was not performed as coil orientation was not common across measures.

AP = anterior-to-posterior induced current. PA = posterior-to-anterior induced current. SICI = short-interval intracortical inhibition. ICF = intracortical facilitation. THT = threshold hunting target. *N*_C_ = number of control datapoints. *N*_ALS_ = number of ALS datapoints.

## Discussion

This study is the first to investigate LICI in ALS using a threshold tracking TMS protocol, and the first to investigate LICI in ALS with multiple induced current directions. Our findings demonstrate that there is no significant difference in LICI, recorded with either PA- or AP-induced current, between those with and without ALS. Both cohorts exhibited similar medians and ranges for all LICI measures. We have also demonstrated that estimates of LICI obtained using PA-induced current on the one hand and AP-induced current on the other are not closely associated. This suggests that the physiological processes mediating the induced effects of these LICI variants are at least partially distinct. We also find no substantial correlation between LICI_PA-50 ms_ and LICI_PA-100–200 ms_. This is in keeping with epidural recordings^12,13^ which indicate that LICI generated at ISIs greater than 100 engages neural circuits in addition to those that mediate LICI elicited using a 50 ms ISI.

### Robust LICI_100–200 ms_ in those with ALS indicates that GABA-B networks do not drive disinhibition in ALS

As aforementioned, a substantial body of evidence indicates that LICI recorded with 100–200 ms ISIs reflects cortical GABA-Bergic inhibition. We observed no statistically significant difference in these measures between those with and without ALS. The corresponding effect sizes were also generally small. We also find little by way of correlation between any of the LICI measures and disease duration, survival time or ALSFRS-R scores. There was thus little evidence to suggest that the physiological processes that mediate the expression of LICI are abnormal for those with ALS of specific stages or severities. Our results do not replicate previous reports that LICI (elicited using ISIs between 100 and 180 ms) is lower in ALS patients than in healthy controls. Similarly, we did not observe an association between the extent of LICI and disease duration, which has been reported previously in two studies that employed cohorts of similar or smaller sizes.^3^ These studies employed fixed intensity/amplitude-based protocols to quantify LICI, as opposed to the threshold tracking protocol employed here. However, prior studies have demonstrated comparability of short afferent inhibition and SICI measures recorded with both approaches. These studies indicate that this difference in protocol is unlikely to explain such a substantial difference in findings, that is, with respect to the impact of ALS.^37,38^ One of these prior studies employed very small sample sizes (*n* = 5–6 people with ALS, *n* = 12 healthy controls),^21^ such that the effects observed may not be representative of the population from which the sample was drawn, especially in the case of a disease as heterogenous as ALS. The other study^3^ employed a similar sample size to that employed here, although a low number of trials (*n* = 7) was used to calculate average MEP amplitudes. Use of less than 20 trials for amplitude-based single and paired-pulse TMS measures has been reported to substantially increase measurement variability.^39,40^ Additionally, this study noted that reduction in LICI in ALS was predominantly due to the 19 individuals with UMN signs. While the disease duration of our participant cohort is similar to that reported in this previous study, we unfortunately do not have access to corresponding UMN scores for comparison to the prior study.

In our prior investigation, which employed ALS and control cohorts that overlapped extensively with those studied here, we found that SICI was lower in those with ALS, than in healthy controls. In respect of SICI_1 ms_, the difference between groups was relatively modest. The differentiation (as expressed by effect size and AUROC) was however greater for SICI_3 ms_. In that study also, we failed to observe a difference between these cohorts in terms of ICF.^1^ Collectively, these findings suggest that the disinhibition thought to be characteristic of ALS is mediated by dysfunction of GABA-Aergic, and not the GABA-Bergic, cortical circuitry which underpins LICI_100–200 ms_.

The cortical silent period (CSP) is another TMS-based measure that is considered to provide insight into cortical GABA-Bergic circuitry. It is quantified as the duration of interruption to voluntary muscle contraction following the MEP evoked by single TMS pulses.^41^ The CSP has been reported to be shortened in ALS in some studies,^32,42-44^ but prolonged or similar^3,45-47^ in others. Investigations of the relationship between CSP and disease duration or severity have reported mixed findings. While some report shortening early in the disease (albeit still within normal range),^44,47^ more have reported no correlation with disease duration^3,44,45^ or clinical rating scales.^32,42,48^ One relatively consistent finding regarding the CSP is that the increase in duration expected at higher stimulation intensities appears to be lost or reduced in ALS, particularly those at later disease stages.^3,43,44,46^

While some control cohort studies find a significant correlation between LICI_100–200 ms_ and CSP (e.g. rho = 0.61^49^), others do not.^50^ The two measures also show distinct influence by muscle fatigue, with CSP duration increasing and LICI reducing with greater fatigue.^51^ While both measures reduce with greater voluntary muscle contraction, there is no significant relationship between these effects.^52^ Additionally, while intrathecal baclofen administration is reported to lengthen the CSP,^53^ intravenous and oral administration is found to have no effect on CSP,^54-56^ with one study demonstrating increased LICI and no change in CSP duration following a 50 mg oral dose within the same cohort.^55^ Notably, CSP is also modulated by GABA-A agonists (e.g. shortening with diazepam^54^), particularly at lower stimulation intensities.^57^ As such, it is possible that the GABA-Aergic dysfunction, rather than GABA-Bergic change, may underpin changes in CSP, or its relationship to stimulation intensity, observed in ALS.

### LICI_PA_ and LICI_AP_ measures do not significantly covary

Here, LICI recorded with PA- and AP-induced current did not correlate. As the effect of baclofen on LICI_AP_ has not yet been reported, the physiological underpinnings of LICI_AP_ are currently more uncertain than those of LICI_PA_. TMS-EEG studies have demonstrated that although similar N100 potentials are evoked for both induced current directions, differences exist in other aspects of the cortical activity observed during LICI_PA_ and LICI_AP_.^58,59^ These findings again support the use of coil orientations beyond the typically-employed PA-induced current alone when utilizing paired-pulse TMS to explore motor cortical function, and dysfunction of motor circuitry in disease. While our findings indicate that LICI is not useful as a biomarker of ALS, it may be useful in the discrimination of ALS from motor pathophysiologies which do affect LICI. However, LICI has yet to be explored in ALS mimic conditions such as multifocal motor neuropathy or primary lateral sclerosis. As such, its value for this purpose remains to be determined.

### Robust LICI_50 ms_ indicates no consistent effect of ALS on underpinning spinal inhibitory circuits

We found no significant difference in LICI_PA-50 ms_ between people with and without ALS, similarly to findings of a prior, smaller study of LICI_PA-55 ms_ in ALS.^21^ The physiological underpinnings of LICI_PA-50 ms_ remain unclear. Epidural recordings have revealed that descending I-waves from the cortex are enlarged, rather than reduced, by conditioning stimuli at this shorter ISI. As such, the reduction in MEP amplitude is proposed to be the result of inhibition of lower, rather than upper, motor neurons.^12^ We did not find any significant correlation between LICI_PA-50 ms_ and LICI_100–200 ms_, despite reliable correlations being observed between LICI_PA-100 ms_ and LICI_PA-150 ms_, between LICI_PA-150 ms_ and LICI_PA-200 ms_ and between LICI_AP-150 ms_ and LICI_AP-200 ms_. This pattern of outcomes is in keeping with the view that LICI_PA-50 ms_ is sensitive to the action of somewhat separate physiological processes. Nonetheless, we found no evidence that these are impaired in ALS or relate to ALS progression.

### Limitations

By nature of requiring a suprathreshold conditioning stimulus, LICI cannot be recorded in individuals with high single pulse thresholds (e.g. >83% if applying conditioning at 120%RMT). As muscle atrophy progresses in ALS, motor thresholds rise, such that it may be impossible to record LICI where other measures requiring subthreshold conditioning (e.g. SICI) can be measured. Additionally, threshold tracking captures inhibition on the basis of an increase in threshold with conditioning. As such, a ceiling effect may occur in those with high motor thresholds and preserved LICI, where the conditioned threshold exceeds 100%MSO. This limited our ability to reliably quantify some LICI measures in some healthy controls and people with ALS. While amplitude-based approaches to LICI measurement do not suffer from this ceiling effect, they instead experience a floor effect which introduces similar limitations.^24^ Such ceiling effects might instead be avoided by selection of smaller target MEP amplitudes for tracking (e.g. 50 µV rather than the conventionally employed 200 µV). Our dataset for LICI_AP_ was limited. This was due to two factors. Due to time constraints the measure was not collected in some healthy controls and people with ALS enrolled towards the end of the study. Time constraints were due to the TMS session also including other TMS and peripheral nerve stimulation measures (e.g. SICI, ICF, CMAP) as well as some people with ALS and healthy controls needing adequate time to take a lunch break after participation before they took part in an afternoon, EEG-based study (separate to that reported here). Also, some other healthy controls and individuals with ALS had RMT_AP_ values such that an intensity of 120% RMT_AP_ exceeded the capacity of the stimulator. As RMT_AP_ is typically greater than RMT_PA_, and LICI protocols employ a suprathreshold conditioning stimulus, it is not always possible to measure LICI_AP_ in individuals where LICI_PA_ can be measured. Correspondingly, our analysis of LICI_AP_ measures has limited statistical power, and small-to-moderate effects of ALS on LICI_AP_ may not have been detected. While measures collected with AP coil orientation can provide additional insights into normal and impaired cortical physiology, the higher stimulation intensities required are likely to limit their widespread use for clinical and research purposes in cohorts with substantially elevated motor thresholds. Additionally, most participants with ALS in this study were concurrently taking riluzole. Riluzole has no appreciable effects on CMAP, RMT or ICF^60,61^ and only transient effects on SICI (both with 1 and 3 ms ISIs), which return to baseline after 12 weeks.^61,62^ The effects of riluzole on LICI have not been reported. However, riluzole shows very little affinity for GABA-B receptors and no evidence has been published to indicate that riluzole affects GABA-Bergic activity.^63^ As all but one of those taking riluzole who were included in this study had been treated for longer than 12 weeks, it is unlikely that this treatment affects our findings. Unfortunately, scores of upper motor neurone signs were not available in this cohort, such that correlations between LICI and these measures cannot be reported, and warrant future investigation.

## Conclusion

In summary, our work demonstrates that LICI is similar in people with and without ALS and does not relate to measures of disease progression or stage. This work also provides novel insights into the relationships between LICI and other paired-pulse TMS measures, and suggests that AP- and PA-based LICI measures reflect the mediation of different physiological processes.

## Supplementary Material

fcag091_Supplementary_Data

## Data Availability

Anonymized data will be made available upon reasonable request to the corresponding author. A manual form of the maximum likelihood PEST protocol employed for threshold tracking is available in the TMS Motor Threshold Assessment Tool, MTAT 2.0, available at: https://www.clinicalresearcher.org/software.htm.
