# Supplementary material for: Long-interval intracortical inhibition is similar in people with and without amyotrophic lateral sclerosis
Source: Brain Commun. 2026 Mar 16;8(2):fcag091. doi: 10.1093/braincomms/fcag091 (PMC13036595; doi:10.1093/braincomms/fcag091)
Supplement: fcag091_Supplementary_Data [file fcag091_supplementary_data.pdf]

Supplementary Materials

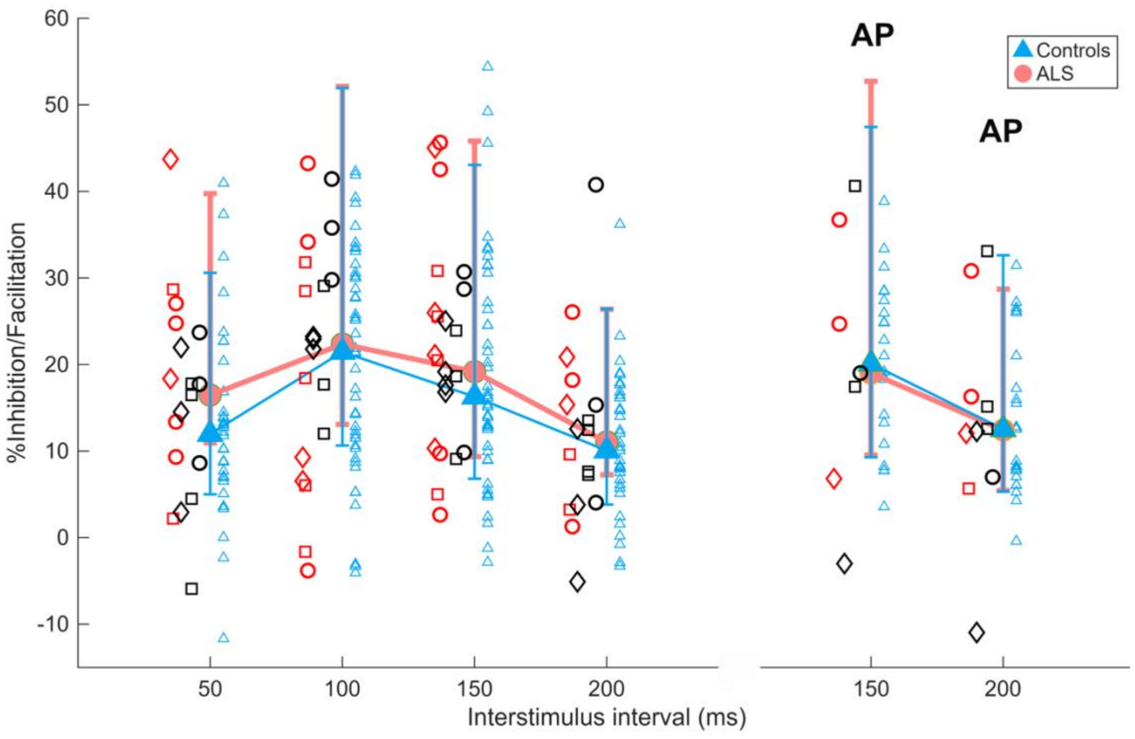

Supplementary Figure 1. Long interval intracortical inhibition median values for healthy controls (blue triangles) and those with spinal-onset ALS of matched side of onset (open circles), unmatched side of onset (squares) or bilateral onset (diamonds), in black for upper limb onset and red for lower limb onset. Error bars illustrate interquartile ranges. Positive y axis values reflect an inhibitory effect of conditioning on the corticospinal tract. Negative y axis values reflect a facilitatory effect of conditioning on the corticospinal tract. PA – Posterior-to-anterior induced current. AP – Anterior-to-posterior induced current.
